# The Impact of Sibling Presence on Motor Competence and Physical Fitness: A Systematic Review

**DOI:** 10.3390/healthcare13233142

**Published:** 2025-12-02

**Authors:** Nerea Blanco-Martínez, Daniel González-Devesa, Pedro Vicente Vila, Antía Esmerode-Iglesias, Carlos Ayán-Pérez

**Affiliations:** 1 Well-Move Research Group, Galicia Sur Health Research Institute (IIS Galicia Sur), Servizo Galego de Saúde-Universidade de Vigo (SERGAS-UVIGO), 36310 Vigo, Spain; n.blanco@uvigo.gal (N.B.-M.); cayan@uvigo.es (C.A.-P.); 2 Departamento de Didácticas Especiáis, Universidade de Vigo, 36310 Vigo, Spain; pachipedrito@edu.xunta.gal; 3 Grupo de Investigación en Actividad Física, Educación, y Salud (GIAFES), Universidad Católica de Ávila, Canteros, 05005 Ávila, Spain; 4 Facultad de Ciencias de la Educación y del Deporte, Universidade de Vigo, 36005 Pontevedra, Spain; a.esmerode7@gmail.com

**Keywords:** siblings, only child, motor competence, fitness, development

## Abstract

**Objective:** This systematic review examined whether siblings act as facilitators or barriers to children’s motor competence and physical fitness. **Methods:** Following PRISMA guidelines, systematic searches were conducted in four databases (Web of Science, Scopus, SPORTDiscus, and MEDLINE/PubMed) up to September 2025. **Results:** Seventeen studies (total n = 116,827) met eligibility criteria. Eleven studies were rated fair quality and the remainder poor. Twelve studies assessed motor competence, four assessed physical fitness, and one addressed both. Children with older siblings often showed better coordination and motor skills, whereas some evidence indicated earlier gross motor development in only children and no consistent differences in fine motor skills. The presence of younger siblings was associated with lower motor skill scores in infants, while older siblings were linked to higher scores. Across motor competence outcomes, the available evidence is concentrated in object control and fine/hand motor skills, with comparatively fewer data on locomotor and stability domains. Regarding physical fitness, siblings generally exerted a positive influence across several dimensions, but these findings are based on a small number of studies, and results for cardiorespiratory fitness are conflicting. **Conclusions:** Given the heterogeneity in ages and measurement tools, along with the predominance of methodological constraints, readers should interpret the findings with caution. In summary, the available evidence suggests that having siblings may be associated with higher motor competence and some aspects of physical fitness, yet the certainty of evidence is limited by heterogeneity (age ranges and measurement tools) and methodological constraints.

## 1. Introduction

Motor competence refers to an individual’s ability to perform various motor skills, supported by factors such as movement quality, motor control, and coordination [1]. It comprises three interrelated subdomains: locomotor skills, involving movements that transport the body from one location to another (e.g., running, hopping, jumping); object control or manipulative skills, referring to actions that handle or project objects with the hands or feet (e.g., throwing, catching, striking, kicking); and stability or balance skills, which relate to maintaining postural control in both static and dynamic contexts [2]. Its development during childhood has a significant positive impact on physical activity levels in adolescence and early adulthood, making it a crucial determinant in fostering an active lifestyle [3]. Given the high prevalence of physical inactivity and sedentary behavior among adults [4], promoting motor competence from an early age is vital [5]. In fact, children with insufficient motor competence often show lower physical activity and fitness levels, underscoring the importance of early interventions [6].

Similarly to motor competence, developing and maintaining satisfactory levels of physical fitness throughout life is essential for preventing disease and reducing mortality [7]. Health-related physical fitness, as defined by Caspersen et al. [8], encompasses five key components—cardiorespiratory fitness, muscular strength, muscular endurance, flexibility, and body composition—all of which contribute to the body’s ability to function efficiently and maintain health [8]. Specifically, cardiorespiratory fitness refers to the capacity of the circulatory and respiratory systems to deliver oxygen during sustained activity [9]. Muscular strength is the ability to exert maximal voluntary force against a resistance, while muscular endurance reflects the capacity to sustain submaximal force over time, both being key components of health-related physical fitness [10]. Flexibility represents the range of motion available at a joint or group of joints [11], and body composition indicates the relative proportion of fat and lean mass in the body [12]. Physical fitness includes a range of physical and physiological attributes that directly influence health [8]. Accordingly, evidence highlights the importance of promoting muscular fitness and cardiovascular endurance from an early age due to their significant health benefits [13,14]. Studies involving children have demonstrated a strong association between cardiorespiratory fitness and cardiometabolic health parameters [7]. Similarly, muscular strength has been recognized as a key indicator of bone health [15]. Furthermore, both cardiorespiratory fitness and muscular strength have been linked to improved quality of life, encompassing physical, psychological, and social well-being [16]. Recent studies have demonstrated that fundamental movement skills (FMSs) such as jumping, hopping, and static balance are strongly associated with health-related fitness components, including muscular strength, endurance, and flexibility, reinforcing the idea that motor competence contributes directly to children’s health and long-term physical development [17].

Notably, physical fitness directly influences an individual’s ability to perform motor tasks [1,8]. Consequently, the scientific literature highlights the importance of achieving adequate physical fitness levels from childhood due to its long-term benefits in adulthood [18,19]. Indeed, although motor competence and physical fitness are interdependent factors, they exhibit a dynamic relationship throughout life, from childhood to adulthood [1]. Through play, children have the opportunity to improve their motor competence and enhance physical fitness [20]. Moreover, developing motor competence in early years fosters improvements in physical fitness, ultimately contributing to better health outcomes later in life [21].

Motor competence and physical fitness are shaped by two primary influences: endogenous factors, reflecting genetic potential, and exogenous factors, encompassing environmental conditions [22,23]. Key exogenous factors include place of residence, family sports tradition, socioeconomic status, and parents’ educational level. Among these, the number of siblings and birth order hold particular significance [24]. Siblings can play an important role in a child’s development through mutual interaction. However, their influence varies depending on whether they assume a positive role as role models and facilitators or a negative role by competing for resources [25].

The scientific literature on the influence of siblings on the development of motor competence and physical fitness, compared to children without older siblings or only children, presents contradictory findings. While some studies report no significant relationships [26], others, such as McHale et al. [27], emphasize that having an older sibling can offer younger children valuable learning opportunities through imitation and observation, potentially enhancing their development. Similarly, regarding birth order, existing evidence remains inconclusive on which position may lead to better performance [28].

In addition, sibling influences may depend on moderators and confounders such as the age gap between siblings, birth order, and the total number of siblings. These variables represent important sources of variation that require systematic attention.

Since siblings can serve as either facilitators or barriers in the development of motor competence and physical fitness, it is crucial to assess their influence, whether positive or negative. To achieve this, systematic reviews are necessary to synthesize the most recent evidence on the topic. However, given the heterogeneity and methodological limitations typically found in this type of study, a systematic review may not be able to establish definitive effects. Instead, it should be positioned as a mapping of the evidence, with the aim of identifying consistencies, contradictions, and gaps in the literature.

To the authors’ knowledge, no systematic review on this subject has been published to date. Therefore, the aim of this study is to conduct a systematic review to identify and critically analyze the best available scientific evidence regarding the influence of siblings on the development of motor competence and physical fitness.

## 2. Materials and Methods

This systematic review was conducted following international recommendations for the development of systematic reviews outlined in the Reporting Items for Systematic Reviews and Meta-Analyses 2020 (PRISMA) guidelines [29]. The protocol for this review was registered on the Open Science Framework (OSF): https://doi.org/10.17605/OSF.IO/MPZAC.

### 2.1. Search Strategy

Systematic searches were conducted in four electronic databases (Web of Science, SPORTDiscus, PubMed, and Scopus) from inception to 24 September 2025. A previous exploratory search was carried out to identify the Boolean search string most appropriate for this review. Table 1 presents the search strategy used in each database

### 2.2. Eligibility Criteria

The studies included in the review met the following inclusion criteria: (1) they specifically evaluated motor competence and/or physical fitness using standardized tests or assessments, and (2) they considered the presence or absence of siblings as a key variable. Qualitative research, as well as studies focusing on children with disabilities or written in languages other than English, Spanish, or Portuguese, were excluded. Studies were not considered for further analysis if, despite assessing motor competence and/or physical fitness in children with and without siblings, they did not establish comparative groups.

### 2.3. Study Selection

Two authors (N.B.-M. and D.G.-D.) reviewed the titles and abstracts of the identified studies to determine their eligibility, independently examined the full text of potentially eligible studies, selected those that met the inclusion criteria, and compared their results to reach an agreement. Finally, based on the selected articles, their references were reviewed, and studies citing them were located through “Google Scholar” to identify potentially includable research with similar characteristics. In the case of any discrepancies in the above steps, advice was sought from a third author (C.A.-P.), and a consensus was reached.

### 2.4. Data Extraction

The data from the included studies were extracted from the original articles by two researchers and reviewed by a third researcher, all of whom have extensive experience in conducting systematic reviews, including the data extraction process. The extracted data included the first author’s name, year of publication, study design, sample characteristics, tests used to measure motor competence and/or physical fitness, and the most relevant results described in the research. Sibling exposure variables included only-child status, presence of older siblings, sibling count, and, when available, age gap, and sex composition. All extracted information was organized into a Microsoft Word table (Table 2). The data extraction process was not blinded, as the names of the selected studies and the titles of the journals in which they were published were known.

### 2.5. Quality Appraisal

The methodological quality of observational studies included in this research was evaluated using the Quality Assessment Tool for Observational Cohort and Cross-Sectional Studies [47]. Risk of bias was assessed independently by at least two researchers and disagreements were resolved by a third investigator. The criteria were evaluated as ‘Yes’, ‘No’, or ‘Other’ (not applicable, not reported, or not determinable). Furthermore, the level of evidence was assessed based on the criteria outlined by Cares-Marambio et al. [48]: good (≥75%), fair (50–75%), and poor (≤50%).

## 3. Results

### 3.1. Study Selection Results

**Figure 1:**
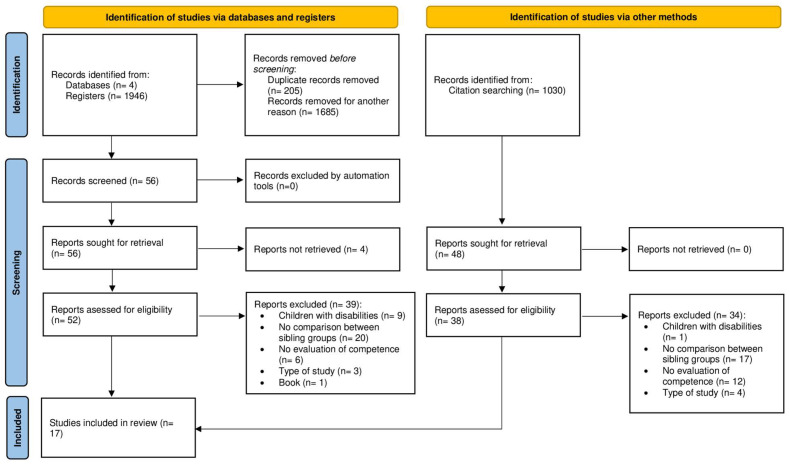
PRISMA (Preferred Reporting Items for Systematic Reviews and Meta-Analyses) study flow diagram.

A total of 1946 records were retrieved from the database search. After removing duplicates, the titles and abstracts of 1741 records were reviewed, and 56 articles were subsequently selected for full-text evaluation. After screening, 13 studies met the inclusion criteria and were included in the systematic review. Finally, additional reports were identified through citations of the selected articles (n = 1030), adding 4 new articles to the selection and concluding the search with a total of 17 articles (Figure 1).

### 3.2. Design and Samples

The reviewed studies were published between 2006 and 2025 and included a total sample of 116,827 participants, with individual study sizes ranging from 55 [31] to 91,619 [36] individuals.

All investigations included mixed-gender samples, with balanced participation (51,079 boys vs. 47,892 girls). Only five studies did not report information about the participants’ gender [30,32,34,35,46]. The participants’ ages varied, with the youngest average age being 4 months [35] and the oldest average age in the range of 7.0–18.0 years [33].

### 3.3. Interventions Characteristics

The studies found focused on comparing groups formed by children with older siblings vs. children without older siblings (n = 7) or groups formed by children with siblings vs. children without siblings (n = 10). Among the total number of investigations reviewed, four aimed to analyze the characteristics of only children. Thus, Krombholz [37] examined the comparison between being an only child and having older siblings, while Jia et al. [36], Rodrigues et al. [41] and Zareian et al. [46] explored the differences between being an only child and having siblings.

In relation to the main outcomes examined, 12 studies evaluated motor competence [30,31,32,34,35,38,39,40,42,43,45,46], 4 analyzed physical fitness [33,36,41,44], and one single study addressed both aspects [37].

To assess motor competence, four studies used standardized batteries: Test Motor Global Rating of Athletic Motor Integration, version 2 (GRAMI-2) [31]; Peabody Developmental Motor Scales—Second Edition (PDMS-2) [40], Motor Competence Assessment (MCA) [42], and the Canadian Agility and Movement Skill Assessment (CAMSA) [34]. One research used field-based test from the Lincoln Oseretsky Motor Development Scale (static and dynamic balance) [46], while three others used field tests focused on specific skills [37,39,43]. Five studies administered questionnaires to be answered by parents or primary caregivers [30,32,35,38,45].

For assessing physical fitness, three studies used field-based tests from batteries such as European Physical Fitness Test Battery (EUROFIT; shuttle-run test) [33] or FitnessGram (Progressive Aerobic Cardiovascular Endurance Run; PACER) [36,41]. Šerbetar et al. [44] used the standardized Presidents Challenge Battery, while Krombholz [37] used the lateral dominance test and Jia et al. [36] the 50 m sprint (anaerobic fitness). Table 2 provides a detailed summary of the main characteristics of the selected studies.

### 3.4. Main Outcomes

#### 3.4.1. Does Having Older Siblings Affect Motor Competence?

The studies analyzed yielded mixed results regarding motor competence. On one hand, the investigations by Chiva-Bartoll & Estevan [31], Krombholz [37] and Schild et al. [45] indicated that children with older siblings showed higher levels of motor coordination, balance, jumping and body motor skills. Moreover, Chiva-Bartoll & Estevan [31] found that when separated by gender, boys with older siblings exhibited better coordination levels compared to their same-gender peers without older siblings, a trend also observed in girls. Similarly, Schild et al. [45] showed that boys with older siblings had superior hand motor skills compared to those without siblings.

On the other hand, Krombholz [38] observed that children without older siblings tend to develop certain gross motor skills at an earlier age, while Cruise & O’Reilly [32] reported that the presence of one or more older siblings in the home might be associated with a higher risk of difficulties in gross motor function. Additionally, both Cruise & O’Reilly [32] and Krombholz [37] found no influence of having an older sibling on fine motor function and manual dexterity, respectively. In contrast, Hayashida & Nakatsuka [35], in their research conducted with 4-month-old infants, found contradictory results depending on the age of the siblings. Babies with siblings under 4 years old showed lower scores in motor skills, while those with siblings aged 5 years or older had higher scores.

#### 3.4.2. Does Having Older Siblings Affect Physical Fitness?

The only studies that focused on fitness found a significant influence of having older siblings on levels of agility and upper body strength [37,44].

#### 3.4.3. Does Having Siblings Affect Motor Competence?

The studies analyzed mostly found that having siblings positively influences motor competence. For instance, Sáez-Sánchez et al. [43] concluded that having a sibling is better than not having one in terms of psychomotor performance. Similarly, Cheng et al. [30] found that, regarding FMS performance, children with siblings outperform only children in manipulative, stability, and locomotor skills. Zareian et al. [46] showed that the birth order had a significant influence on static balance, the second children was higher than the first and only children. Similarly, Rebelo et al. [40] conducted research across different age groups and found that children aged 12–23 months with older siblings showed better fine motor skills. Moreover, children aged 24–35 months with siblings scored higher in motor skill development across various areas, such as posture, locomotion, object manipulation, visual motor integration, gross motor skills, and fine motor skills. In the 36–48-month group, having a sibling was associated with better locomotor development, object manipulation, visual motor integration, and gross motor skills. Although three studies did not identify significant differences between children with or without siblings [34,39,42], the study by Rodrigues et al. [42] observed that children with siblings were in a higher percentile for both the total score and all subscales of the motor battery.

#### 3.4.4. Does Having Siblings Affect Physical Fitness?

Three of the investigations that analyzed physical fitness found that having siblings was significantly associated with higher levels of muscular strength and endurance [41,44] and anaerobic fitness [36]. Only González-Devesa et al. [33] found no association between the number of siblings and agility levels.

Regarding cardiorespiratory fitness, the results were mixed. Rodrigues et al. [41] reported a positive influence of having siblings on PACER performance, while Jia et al. [36] found no significant differences in PACER scores between only children and those with siblings.

### 3.5. Quality Appraisal Results

The methodological quality of the studies included in this review was rated as fair for eleven investigations [30,32,33,34,35,36,37,39,41,45,46] and poor for the remaining studies. While all investigations clearly described the objectives of the paper and provided detailed descriptions of the outcomes, several limitations were identified. Among the most common were the lack of exposure assessment prior to outcome measurement, the absence of a sufficient timeframe to observe an effect, and the lack of blinding of outcome assessors, among others (Table 3).

## 4. Discussion

The aim of this review was to analyze the influence that the presence of siblings may have, either as a facilitator or a barrier, on the development of motor competence and physical fitness. An acceptable number of studies were identified, although they showed high heterogeneity and were predominantly of moderate methodological quality. Despite the methodological limitations, several findings are noteworthy, as they provide relevant information on the role that siblings might play in the developmental process, whether facilitating or hindering it.

The results obtained do not allow for a clear conclusion regarding the benefits or drawbacks of having an older sibling compared to not having older siblings on the development of motor coordination. While two studies highlight possible benefits of having an older sibling for the development of motor coordination, body motor skills and hand motor skills [31,45], two others suggest that the presence of an older sibling could pose a risk and hinder the development of gross motor skills [32,38]. This positive or negative impact on motor skill development may be influenced by factors such as the age of the older sibling, as noted by [35] in their study on infants, as well as the gender of the siblings, according to Chiva-Bartoll & Estevan [31].

Indeed, the age gap has been observed to significantly influence the relationships established between siblings [49], while gender differences may also affect the acquisition and development of linguistic skills, with a positive influence observed from having older sisters [50]. Additionally, the siblings’ intelligence quotient could play an important role, as older siblings with higher intelligence quotients might be more likely to cooperate in the development of their younger siblings [51]. However, the studies reviewed did not take this variable into account, making it impossible to explore this aspect further.

Regarding the influence of older siblings on physical fitness parameters, Krombholz [37] and Šerbetar et al. [44] reported positive results supporting this relationship in various aspects. Specifically, the positive impact of having an older sibling on agility levels may be noteworthy, as both studies highlighted this association. In contrast, simply having siblings, regardless of their age, was not found to be a significant factor according to González-Devesa et al. [33]. This possible positive effect is also reflected in health aspects such as body composition, as the presence of an older sibling is associated with better body mass index values [52], whereas their absence is linked to higher body mass index levels [53]. Additionally, having an older sibling appears to positively influence participation in sports activities [54]. In this sense, motor competence and physical fitness should also be understood as protective factors for health, since evidence shows that greater body fat is associated with less strength, impaired balance, and a greater risk of chronic diseases and functional impairment in adulthood [55]. Nevertheless, the evidence on physical fitness remains limited, with only weak signals in anaerobic capacity, muscular endurance, and agility, and conflicting findings in cardiorespiratory fitness. In this regard, it should be noted that the study by Jia et al. [28] included a sample size of more than 90,000 children, whereas Rodrigues et al. [46] analyzed only 500. Therefore, current evidence does not support a definite effect of sibling presence on aerobic fitness.

The included studies suggest that having a sibling may promote the development of motor competence, specially in object control and fine motor domains, and physical fitness compared to not having one [30]. However, in the study by González-Devesa et al. [34], the presence of siblings did not have a significant effect on motor competence as assessed through the CAMSA. The presence of a sibling, whether older or younger, seems to be associated with physical activity levels, sports experience [56,57], and the acquisition of certain motor skills [58]. This positive influence could be related to the constant interaction and mutual imitation that often occurs between siblings [27], which can enhance the development of motor abilities and overall physical fitness. However, it is important to recognize that the manifestation of this association may be influenced by specific family environment factors, as seen in the development of basic skills such as crawling and walking [25]. In any event, it should be noted that some of the reviewed studies exhibited methodological flaws, including exposure assessment prior to outcome measurement, insufficient timeframes to observe an effect, and a lack of blinding of outcome assessors. Therefore, caution is warranted when interpreting the purported positive impact of having a sibling on motor competence and physical fitness.

Scientific evidence has demonstrated that poor physical fitness during adolescence in only children significantly increases the likelihood of developing overweight and obesity, which, in adulthood, translates into a higher risk of mortality [59]. Additionally, various studies have indicated that growing up without siblings is associated with higher blood pressure levels [60]. In this research, studies analyzing differences in physical health parameters between only children and those with siblings have reported mixed results regarding aerobic fitness. Although Rodrigues et al. [41] reported a positive influence of having siblings on performance, Jia et al. [36] found no differences in scores between only children and those with siblings. This discrepancy may be due to differences in the age of the samples, so other factors, such as sports practice or training capacity, could come into play [61].

That said, it is crucial to interpret these associations with caution. Some studies emphasize that motor development does not depend exclusively on being an only child or having siblings, highlighting the importance of other determining factors, such as the child’s individual characteristics, the specific dynamics of the family environment, and various elements unique to each family [62,63].

### 4.1. Limitations

This appears to be the first systematic review focused on the role of having siblings in the development of motor competence and physical fitness. However, the results obtained do not conclusively confirm the facilitating effect of having an older sibling on the development of the younger sibling. Despite the originality of the study, there are some limitations that should be considered. Firstly, most of the studies on motor competence primarily focused on manual dexterity and fine motor skills, without exploring other important aspects of motor development, such as locomotion. Secondly, there was a limited number of studies investigating the influence of having siblings on health-related physical fitness parameters. In addition, one very large study may have disproportionately influenced the findings. Thirdly, other contextual and environmental factors—such as opportunities for practice, participation in sports, genetic characteristics, and home environment—along with differences in age categories, may have influenced the results, making it difficult to isolate the effect of sibling presence. Factors such as age gap, sex constellation, birth order, family sport culture, and socioeconomic status represent additional confounding variables that were generally not assessed, further complicating the ability to draw robust conclusions

Fourthly, heterogeneity in the tools used to assess motor competence and in the age ranges of participants further complicates comparisons across studies. In addition, many of the included studies did not provide sufficient detail regarding age bands, measurement types, or motor subdomains, and in some cases, reporting was highly heterogeneous, which prevented further subgroup analysis.

Lastly, the exclusion of gray literature and the restriction to studies published in certain languages may have narrowed the scope of the identified research, potentially overlooking relevant evidence that could have enriched the review and influencing the comprehensiveness of the results. These limitations justify interpreting the conclusions at the level of association rather than as generalizable or causal

### 4.2. Implications for Practice

The synthesis of evidence highlights that children’s motor competence is shaped by environmental and family factors, with siblings potentially playing a central role. Practitioners in educational and sport settings may consider that motor development is not only biological maturation but is also shaped by context.

The review suggests that children with siblings may show certain advantages in motor competence compared to only children, as older siblings can provide models to imitate and enrich the home environment. For children without siblings, schools and community programs could create peer-mediated contexts that may reproduce the benefits of sibling interactions. Motor competence is closely linked to health-related fitness [17] and cognitive development [64], which suggests that cooperative motor tasks and mixed-ability grouping in physical education could represent promising approaches warranting further investigation.

Children with higher motor proficiency are more likely to sustain physical activity and healthier lifestyles in adolescence and adulthood. This evidence underscores the importance of early interventions in preschool and primary school that emphasize group-based activities and foster positive peer interactions, as these stages are critical for establishing the foundations of lifelong health and participation in physical activity.

## 5. Conclusions

Preliminary scientific evidence suggests that having a sibling may act as a facilitator in certain aspects of motor competence development, especially object control and fine motor skills, whereas the evidence for health-related fitness remains limited and inconsistent. However, the results of this review do not definitively determine whether having an older sibling has a significantly greater positive effect, as other contextual and environmental factors may also be influencing the outcomes. A relevant aspect that deserves greater attention in future research is that most studies were focused on motor competence, while fewer addressed physical fitness, leaving an important gap in the understanding of physical development and health. Moreover, within the field of motor competence, studies tended to focus primarily on manipulative skills, highlighting the need for more comprehensive analyses that include other skill domains. In addition, future studies should use standardized tools and incorporate mediation analyses to examine whether social and contextual variables —including age gap, sex constellation, birth order, practice opportunities, participation in sports activities, and genetic characteristics—influence these outcomes, which would allow for a clearer understanding of their relationship with the presence or absence of siblings.

## Figures and Tables

**Table 1:** Full search strategy for each database with arguments presented as they were used.

Database	Search Strategy
Web of Science	TI = ((“sibling*” OR “brother*” OR “sister*” OR “twin*”) AND (“psychomotor performance” OR “motor activity” OR “motor competence” OR “motor proficiency” OR “motor ability” OR “motor performance” OR “movement competence” OR “gross motor competence” OR “fundamental movement skills” OR “motor skills” OR “physical fitness”))
Scopus	TITLE-ABS-KEY ((“sibling*” OR “brother*” OR “sister*” OR “twin*”) AND (“psychomotor performance” OR “motor activity” OR “motor competence” OR “motor proficiency” OR “motor ability” OR “motor performance” OR “movement competence” OR “gross motor competence” OR “fundamental movement skills” OR “motor skills” OR “physical fitness”))
SportDiscus	(“sibling*” OR “brother*” OR “sister*” OR “twin*”) AND (“psychomotor performance” OR “motor activity” OR “motor competence” OR “motor proficiency” OR “motor ability” OR “motor performance” OR “movement competence” OR “gross motor competence” OR “fundamental movement skills” OR “motor skills” OR “physical fitness”)
MEDLINE/PubMed	(“sibling*”[Title] OR “brother*”[Title] OR “sister*”[Title] OR “twin*”[Title]) AND (“psychomotor performance”[Title] OR “motor activity”[Title] OR “motor competence”[Title] OR “motor proficiency”[Title] OR “motor ability”[Title] OR “motor performance”[Title] OR “movement competence”[Title] OR “gross motor competence”[Title] OR “fundamental movement skills”[Title] OR “motor skills”[Title] OR “physical fitness”[Title])

**Table 2:** Descriptive characteristics of he included studies.

Fist Author (Year)	Sample	Objective	Measure	Results
Cheng et al. (2025) [30]	**Participants (n, sex):** 6200 (NR) **Age (mean ± SD):** 2–6 years **With/without siblings (n, sex, age):** Children with 1 sibling (n = 2648, NR, NR) Children with 2 siblings (n = 317, NR, NR) Children with 3 siblings (n = 35, NR, NR) Children without sibling (n = 3200, NR, NR)	To explore the impact of different family background on children’s physical activity.	**Outcomes:** FMS - Manipulative motor skills. - Stability motor skills. - Locomotor motor skills. **Measurement tool:** Self-reported questionnaire fulfilled by primary caregivers.	Performance on the FMS scale of children in families with siblings was better than that of children in families with only one child. - Children with siblings had a higher level of manipulative skills than children without siblings (F-stat = 57.73; *p* < 0.001). - Children with siblings had a higher level of stability skills than children without siblings (F-stat = 60.46; *p* < 0.001). - Children with siblings had a higher level of locomotor skills than children without siblings (F-stat = 92.88; *p* < 0.001).
Chiva-Bartoll & Estevan (2019) [31]	**Participants (n, sex):** 55 (23F; 22M) **Age (mean ± SD):** 8.51 ± 0.31 years **With/without siblings (n, sex, age):** Children with older siblings or siblings of the same age (n = 37, NR, NR) Children without older or same-age siblings (n = 18, NR, NR)	To analyze the relationship between having or not having older siblings and the level of motor coordination.	**Outcome:** Motor Coordination. **Measurement tool:** GRAMI-2: - 30 m running. - Medicine ball throw 1 kg. - 7 m jumping on one leg. - Lateral jumps. - One-way run. - Displacement on support.	For the total sample, it was observed that participants with older siblings or siblings of the same age had a higher level of coordination than those without (t = 4.73; *p* = 0.01). Children with siblings had a higher level of motor coordination than children without siblings (GRAMI-2 total score: 53.97 ± 3.67; vs. 47.91 ± 3.80; *p* = 0.002). Girls with siblings had a higher level of motor coordination than girls without siblings (GRAMI-2 total score: 49.44 ± 4.33 vs. 41.59 ± 5.55; *p* = 0.017).
Cruise & O’Reilly. (2014) [32]	**Participants (n, sex):** 10,748 last born infants, NR **Age:** 9 months **With/without siblings (n, sex, age):** Children with 1 sibling (n = 3584, NR, NR) Children with 2 or more siblings (n= 2589, NR, NR) Children without sibling (n = 4571, NR, NR)	To examine the influence of parents, siblings, and aspects of non-parental care on infant development.	**Outcomes:** Gross Motor Function. Fine Motor Function. **Measurement tool:** Self-reported questionnaire fulfilled by primary caregivers: - Ages and Stages Questionnaire.	The presence of one or more older siblings in the household increased the risk of failure in gross motor function: - OR for one sibling: 1.61; (95% CI 1.40–1.85). - OR for two or more siblings: 1.76; (95% CI 1.50–2.08). The presence of one or more siblings in the home does not increase the risk of failure in fine motor skills. - OR for one sibling: 0.99; (95% CI 0.84–1.17). - OR for two or more siblings: 1.01; (95% CI 0.83–1.23).
González-Devesa et al. (2024) [33]	**Participants (n, sex):** 579 (271F; 308M) **Age (range; mean ± SD):** 7–18; 12.17 ± 2.91 years **With/without siblings (n, sex, age):** Children with 1 sibling (n = 265, NR, NR) Children with 2 siblings (n = 161, NR, NR) Children with 3 or more sibling (n = 32, NR, NR) Children without sibling (n = 121, NR, NR)	To confirm whether the 2D:4D digit ratio and having siblings are independent factors that affect motor development, as assessed through agility both in children and adolescents.	**Outcomes:** Agility. **Measurement tool:** Field-based test: 10 × 5 shuttle run test from EUROFIT battery.	No association was found between 10 × 5 m shuttle run test and number of siblings (Rho = −0.074; *p* = 0.076).
González-Devesa et al. (2025) [34]	**Participants (n, sex):** 432 (NR) **Age (range; mean ± SD):** 6–11; 8.81 ± 1.8 years 12–16; 13.52 ± 1.22 years **With/without siblings (n, sex, age):** Children with older siblings or siblings of the same age (n = 149, NR, NR) Children without older or same-age siblings (n = 283, NR, NR)	To investigate the influence of relative age and the effects of the presence of siblings on the motor competence of children and adolescents.	**Outcomes:** Motor competence. **Measurement tool:** CAMSA	The presence of siblings did not have a statistically significant effect on CAMSA performance (*p* = 0.697; β = −0.019).
Hayashida & Nakatsuka (2013) [35]	**Participants (n, sex):** 318, NR **Age:** 4 months **With/without siblings (n, sex, age):** Babies with siblings < 4 years (n = 108, NR, NR) Babies with siblings ≥ 5 years (n = 35, NR, NR) Babies without siblings (n = 141, NR, NR)	To assess correlations between various factors and the physical development of 4-month-old infants.	**Outcomes:** - Motor Skills. - Motor Score. **Measurement tool:** Self-reported questionnaire fulfilled by parents: - KIDS-A	Babies with siblings < 4 years old had lower scores in KIDS-A compared to those without siblings (10.0 [5–14] vs. 11.0 [6–14]; *p* < 0.001). No differences were found in KIDS-A scores when babies with siblings ≥ 5 years and babies without siblings were compared (11.0 [7–14] vs. 11.0 [6–14]; *p* > 0.05). Babies with siblings aged ≥5 years had higher scores in KIDS-A compared to babies with siblings < 4 years old (11.0 [7–14] vs. 10.0 [5–14]; *p* < 0.05). No intergroup differences were found in KIDS-A in any of the groups (none = 10.0 [2–13]; <4 years = 10.0 [4–13]; ≥5 years = 10.0 [6–13]; *p* = 0.656).
Jia et al. (2022) [36]	**Participants (n, sex):** 91,619 (44320F; 47299M) **Age (mean ± SD):** 10.4 ± 0.7 **With/without siblings (n, sex, age):** Children with siblings (n = 62,988, NR, NR) Only children (n = 28,631, NR, NR) 50 m sprint: Children with siblings (n = 55,535) Only children (n = 25,556) PACER test: Children with siblings (n = 55,614) Only children (n = 25,198)	To analyze whether the status of “only child” affects school performance (including physical health).	**Outcome:** Physical fitness. **Measurement tool:** Field-based tests: - 50 m sprint. - PACER test.	Children with siblings achieved significantly better 50 m sprint times compared to only children (*p* < 0.001). No significant differences in cardiorespiratory fitness were observed between only children and those with siblings (*p* > 0.05).
Krombholz (2006) [37]	**Participants (n, sex):** 1194 (556F; 638M) **Age interval:** 43–84 months	To analyze the relationship of three dimensions of physical performance with age, sex, birth order, participation in sport activities, and socioeconomic status and with cognitive performance in preschool children.	**Outcomes:** Motor Coordination. **Measurement tool:** Field-based tests: - Forward balancing. - Hopping on the right and left foot. - Backward balancing. - Lateral jump. **Outcome:** Physical fitness. **Measurement tool:** Field-based tests: - Standing broad jump. - Shuttle run test. - Hanging task. **Outcome:** Manual dexterity. **Measurement tool:** Field-based test: - Lateral Dominance Test.	Children with older siblings obtained significantly better values than only children in the following: - Balance. - Lateral jump. - Shuttle run. - Right foot hop. - Arm hang.
Krombholz (2023) [38]	**Participants (n, sex):** 3200 (1568F; 1578M; 54 no gender information) **Age interval:** 10–14 months **With/without siblings (n, sex, age):** Children with older siblings (n = 960, NR, NR) Children without older siblings (n = 2240, NR, NR)	To assess the motor development of children without siblings compared to children who had an older sibling in the first two years of life.	**Outcome:** Motor development. **Measurement tool:** Self-reported questionnaire fulfilled by parents on the mastering of 18 motor skills: - 14 gross motor skills: bring hands together, lift head on stomach, roll to prone, roll to supine, sitting with support, sit up unsupported, belly crawl, hands and knees crawling, standing up with support, standing with help, walk sideways with hold, pull to stand, walking alone, walking alone and safely. - 4 hand motor skills: grasp after things, pass an object to the other hand, tweezer grip, pincer grip.	Children without older siblings mastered earlier than children with older siblings the following gross motor skills: Bring hands together earlier (72 ± 32 vs. 67 ± 32; *p*: 0.01). Children without older siblings mastered earlier than children with older siblings the following manual skills: - Grasp after thing (101 ± 27 vs. 95 ± 34; *p* = 0.01). - Pass an object (186 ± 59 vs. 163 ± 51; *p* = 0.01). - Tweezer grip (215 ± 61 vs. 200 ± 68; *p* = 0.01). - Pincer grip (250 ± 66 vs. 233 ± 51; *p* = 0.01).
Lopes & Monteiro (2021) [39]	**Participants (n, sex):** 181 (84F; 97M) **Age ± SD:** 6.10 ± 0.47 years	To assess the effect of somatic and selected socio-cultural factors on motor competence of five to six-year-old children.	**Outcomes:** Motor competence. **Measurement tools:** Field-based tests: Tennis ball throw for distance. Speed run 15 m. Standing long jump.	No significant association between having or not having siblings and motor competence was observed.
Rebelo et al. (2020) [40]	**Participants (n, sex):** 405 (206F; 199M) **Groups of age (n, age ± SD):** From 12 to 23 months (n = 107, age = 18.79 ± 3.73) From 24 to 35 months (n = 153, age = 28.07 ± 3.35) From 36 to 48 months (n = 145, age = 39.31 ± 3.56) **With/without siblings (n, sex age ± SD):** Children with siblings (n = 199, NR, age = 30.61 ± 8.78) Children without siblings (n = 208, NR, age = 30.70 ± 8.67)	To verify whether the presence of siblings influenced the motor skills development of children in the first 48 months of life.	**Outcomes:** Motor skill development. **Measurement tool:** PDMS-2: Postural skills, locomotion skills, object manipulation skills, fine manipulation skills, visuo-motor integration skills. Motor quotients (global motricity and fine motricity).	Group of age from 12 to 23 months: Children with siblings showed higher levels than children without siblings in the following: - Fine motricity (98.50 ± 7.77 vs. 93.90 ± 9.09; *p* = 0.005). Group of age from 24 to 35 months: Children with siblings showed higher levels than children without siblings in the following: - Postural skills (12.11 ± 1.83 vs. 11.42 ± 1.19; *p* = 0.001). - Locomotion skills (9.11 ± 1.52 vs. 8.29 ± 1.74; *p* = 0.001). - Object manipulation skills (9.22 ± 1.60 vs. 8.61 ± 2.06; *p* = 0.039). - Visuo-motor integration skills (10.28 ± 1.98 vs. 8.51 ± 1.91; *p* < 0.001). - Global motricity (101.74 ± 8.21 vs. 94.62 ± 12.19; *p* < 0.001). - Fine motricity (100.57 ± 8.71 vs. 96.24 ± 8.21; *p* = 0.001). Group of age from 36 to 48 months: Children with siblings showed higher levels than children without siblings in the following: - Locomotion skills (9.39 ± 1.08 vs. 8.83 ± 1.06; *p* = 0.014). - Object manipulation skills (9.44 ± 1.62 vs. 8.32 ± 1.27; *p* < 0.001). - Visuo-motor integration skills (11.54 ± 2.31 vs. 10.71 ± 1.89; *p* = 0.033). - Global motricity (103.41 ± 7.66 vs. 99.89 ± 7.98; *p* < 0.001).
Rodrigues et al. (2020) [41]	**Participants (n, sex):** 540 (270F; 270M) **Age interval:** 7–15 years **With/without siblings (n, sex, age):** Children with siblings (n = 399, 202F; 197M, NR) Children without siblings (n = 141, 70F; 71M, NR) Age groups with sibling vs. only child: - From 7 to 9 years (110 vs. 36) - From 10 to 12 years (46 vs. 19) - From 13 to 15 years (46 vs. 15)	To examine if being an only child is associated with negative differences on somatic growth and physical fitness compared to being a child with siblings.	**Outcomes:** Physical fitness. **Measurement tools:** Field-based tests: - Handgrip strength. - Flexed arm hang. - 60 s sit-ups. - Standing long jump. - 10 m shuttle run test. - PACER test.	Children with siblings had better values than only child in the following: - Flexed arm hang (F-stat = 3.989; *p* = 0.046). - 60 s sit-ups (F-stat = 9.355; *p* = 0.002). - 10 m shuttle run test (F-stat = 6.166; *p* = 0.013). - PACER test (F-stat = 4.636; *p* = 0.032).
Rodrigues et al. (2021) [42]	**Participants (n, sex):** 161 (74F; 87M) **Age interval:** 3–6 years **With/without siblings (n, sex, age ± SD):** Children with siblings (n = 125, 54F; 71M, age = 4.7 ± 0.79) Children without siblings (n = 34, 19F; 15M, age = 4.6 ± 0.70)	To evaluate the effect of siblings on the three dimensions of motor competence (stability, locomotor and manipulative).	**Outcomes:** Motor competence. **Measurement tool:** MCA: - Stability skills: lateral jumps, shifting platforms). - Locomotor skills (standing long jump, 10 m shuttle run). - Manipulative skills (ball kicking velocity, ball throwing velocity).	Children with siblings show a higher percentile average for total MCA and all subscales, nevertheless not statistically significant. No statistically significant differences in the MCA total classification were observed, indicating that a higher percentage of children with siblings placed in the higher proficiency group (37% vs. 18%) and a lower percentage in the average proficiency group (30% vs. 50%). The strength of the association was low (Cramer’s V = 0.20). No other statistically significant differences were observed for all other MCA subscales.
Sáez-Sánchez et al. (2021) [43]	**Participants (n, sex):** 215 (101F; 114M) **Age interval (mean ± SD):** 3–6 years (3.98 ± 0.82) **Groups of age:** From 3 years to 3 years and 11 moths (n = 70) From 4 years to 4 years and 11 moths (n = 83) > 5 years (n = 62) **With/without siblings (n, sex, age):** - Children with siblings (n = 132, NR, NR) - Children without siblings (n = 83, NR, NR)	To ascertain whether there are relations of dependency between psychomotor performance in early childhood education and the number of siblings.	**Outcomes:** Pyschomotor Performance. **Measurement tool:** Checklist of Psychomotor Activities - Physical-motor aspects: laterality, dynamic coordination, balance, motor execution, tonal-postural control, respiratory control. - Perceptual-motor aspects: scheme and body image, visual-motor dissociation, spatial orientation and structuration.	Having siblings had a significant impact both on physical (U = 3795.5; *p* < 0.01; d = 0.53) and perceptual (U = 4085.5; *p* < 0.01; d = 0.43) motor aspects.
Šerbetar et al. (2021) [44]	**Participants (n, sex):** 108 (67F; 41M) **Age interval (mean ± SD):** 9–10 years (9.45 ± 0.50) **With/without siblings (n, sex, age):** - Children with siblings (n = 55, NR, NR) - Children without siblings (n = 53, NR, NR)	To determine whether children with an older siblings differ from the children without older siblings in physical fitness and body measures.	**Outcomes:** Motor fitness. **Measurement tool:** Presidents Challenge Battery - Pull-ups. - Curl-ups. - V-Sit and Reach. - Shuttle run. - One-mile run. - Handgrip strength.	Children with older siblings showed higher levels than children without siblings in the following: - Pull-ups (F-stat = 5.74; *p* = 0.018). - Shuttle run (F-stat = 4.63; *p* = 0.034).
Schild et al. (2022) [45]	**Participants (n, sex):** 778 (378F; 400M) **Age (mean ± SD; range):** 2.67 ± 1.78 years < 2 years (n = 349) 2–6 years (n = 429) **With/without siblings (n, sex, age):** With siblings (n = 225, NR, NR) Without siblings (n = 269, NR, NR)	To explore environmental and individual factors that are associated with child development and to investigate whether the strength of these associations differs according to the age of the children.	**Outcomes:** Body Motor Skills. Hand Motor Skills. **Measurement tool:** Self-reported questionnaire fulfilled by parents: - Development Test for Children between 6 Months and 6 Years.	Children with older siblings had better levels than children without siblings in the following: - Body motor skills (B = 0.55 [0.06 to 1.04]; *p* = 0.029). - Hand motor skills (B = 0.55 [0.08 to 1.03]; *p* = 0.024).
Zareian et al. (2014) [46]	**Participants (n, sex):** 94, NR **Age interval:** 9–11 years	To study the role of birth order and birth weight in the static and dynamic balance of boys aged 9–11 years old.	**Outcomes:** Motor competence: - Static and dynamic balance. **Measurement tool:** Lincoln Oseretsky Motor Development Scale.	Birth order had a significant influence on static balance (F-stat = 53.231; *p* = 0.001). Static balance for second children was higher than the first and only children at all levels.

CAMSA = Canadian Agility and Movement Skill Assessment; CI = Confidence Interval; EUROFIT = European Physical Fitness Test Battery; F = Female; FMS = Fundamental Motor Skills; GRAMI-2 = Global Rating of Athletic Motor Integration, version 2; KIDS-A = KIDS Type A test; M = Male; MCA = Motor Competence Assessment; NR = Not Reported; OR = Odds Ratio; PACER = Progressive Aerobic Cardiovascular Endurance Run; PDMS-2 = Peabody Developmental Motor Scales, 2nd ed.; SD = Standard Deviation.

**Table 3:** Quality appraisal of included studies.

	1	2	3	4	5	6	7	8	9	10	11	12	13	14	Overall Rating
Cheng et al. (2025) [30]	+	+	+	+	-	-	-	-	+	-	+	-	NA	+	Fair
González-Devesa et al. (2025) [34]	+	+	-	+	-	-	-	+	+	-	+	-	NA	+	Fair
González-Devesa et al. (2024) [33]	+	+	+	+	-	-	-	+	+	-	+	-	NA	-	Fair
Krombholz (2023) [38]	+	-	-	-	+	-	-	+	+	-	+	-	NA	-	Poor
Schild et al. (2022) [45]	+	+	+	+	+	-	-	-	+	-	+	-	NA	+	Fair
Jia et al. (2022) [36]	+	+	+	+	+	-	-	+	+	-	+	-	NA	+	Fair
Sáez-Sánchez et al. (2021) [43]	+	+	-	+	-	-	-	+	+	-	+	-	NA	-	Poor
Šerbetar et al. (2021) [44]	+	-	+	+	-	-	-	+	-	-	+	-	NA	-	Poor
Rodrigues et al. (2021) [42]	+	-	+	+	-	-	-	+	+	-	+	-	NA	-	Poor
Lopes & Monteiro (2021) [39]	+	+	+	+	-	-	-	+	+	+	+	-	NA	+	Fair
Rodrigues et al. (2020) [41]	+	+	+	-	-	-	-	+	+	+	+	-	NA	-	Fair
Rebelo et al. (2020) [40]	+	-	+	+	-	-	-	+	+	-	+	-	NA	-	Poor
Chiva-Bartoll & Estevan (2019) [31]	+	-	+	+	-	-	-	-	+	-	+	-	NA	-	Poor
Zareian et al. (2014) [46]	+	+	+	+	-	-	-	+	+	-	+	-	NA	-	Fair
Hayashida & Nakatsuka (2014) [35]	+	+	+	+	+	-	-	-	+	-	+	-	NA	-	Fair
Cruise & O’Reilly (2014) [32]	+	+	+	+	+	-	-	-	+	-	+	-	NA	-	Fair
Krombholz (2006) [37]	+	-	+	+	-	-	-	+	+	+	+	-	NA	-	Fair
% studies meeting the criterion	100	65	82	88	29	0	0	71	94	18	100	0	NA	29	

1. Was the research question or objective in this paper clearly stated? 2. Was the study population clearly specified and defined? 3. Was the participation rate of eligible persons at least 50%? 4. Were all the subjects selected or recruited from the same or similar populations (including the same time period)? Were inclusion and exclusion criteria for being in the study prespecified and applied uniformly to all participants? 5. Was a sample size justification, power description, or variance and effect estimates provided? 6. For the analyses in this paper, were the exposure(s) of interest measured prior to the outcome(s) being measured? 7. Was the timeframe sufficient so that one could reasonably expect to see an association between exposure and outcome if it existed? 8. For exposures that can vary in amount or level, did the study examine different levels of the exposure as related to the outcome (e.g., categories of exposure, or exposure measured as continuous variable)? 9. Were the exposure measures (independent variables) clearly defined, valid, reliable, and implemented consistently across all study participants? 10. Was the exposure(s) assessed more than once over time? 11. Were the outcome measures (dependent variables) clearly defined, valid, reliable, and implemented consistently across all study participants? 12. Were the outcome assessors blinded to the exposure status of participants? 13. Was loss to follow-up after baseline 20% or less? 14. Were key potential confounding variables measured and adjusted statistically for their impact on the relationship between exposure(s) and outcome(s)? “NA” indicates “not applicable,” reflecting the specific design of the studies. “+” indicates that the study meets the quality criterion; “-” indicates that it does not.

## Data Availability

No new data were created or analyzed in this study. Data sharing is not applicable to this article.

## References

[1] Utesch T., Bardid F., Büsch D., Strauss B. (2019). The Relationship Between Motor Competence and Physical Fitness from Early Childhood to Early Adulthood: A Meta-Analysis. Sports Med..

[2] Gallahue D.L., Ozmun J.C., Goodway J. (2012). Understanding Motor Development: Infants, Children, Adolescents, Adults.

[3] Carson Sackett S., Edwards E.S. (2019). Relationships among Motor Skill, Perceived Self-Competence, Fitness, and Physical Activity in Young Adults. Hum. Mov. Sci..

[4] Denche-Zamorano Á., Mendoza-Muñoz M., Carlos-Vivas J., Muñoz-Bermejo L., Rojo-Ramos J., Pastor-Cisneros R., Giakoni-Ramírez F., Godoy-Cumillaf A., Barrios-Fernandez S. (2022). A Cross-Sectional Study on Self-Perceived Health and Physical Activity Level in the Spanish Population. Int. J. Environ. Res. Public Health.

[5] Estevan I., Barnett L.M. (2018). Considerations Related to the Definition, Measurement and Analysis of Perceived Motor Competence. Sports Med..

[6] Lopes N., Jacinto M., Monteiro D., Matos R., Sergio J.I. (2025). Effects of a Twelve-Week Sports Program on Motor Competence 2 in Children Aged 6 to 10 Years Old—A Study Protocol. Healthcare.

[7] Garciá-Hermoso A., Ramírez-Vélez R., Garciá-Alonso Y., Alonso-Martínez A.M., Izquierdo M. (2020). Association of Cardiorespiratory Fitness Levels during Youth with Health Risk Later in Life: A Systematic Review and Meta-Analysis. JAMA Pediatr..

[8] Caspersen C., Powell K., Christenson G. (1985). Physical Activity, Exercise, and Physical Fitness: Definitions and Distinctions for Health-Related Research. Public Health Rep..

[9] Raghuveer G., Hartz J., Lubans D., Wiltz J., Mietus-snyder M., Perak A., Baker-smith C., Pietris N., Edwards N. (2020). Cardiorespiratory Fitness in Youth—An Important Marker of Health: A Scientific Statement From the American Heart Association. Circulation.

[10] Ortega F.B., Ruiz J.R., Castillo M.J., Sjöström M. (2008). Physical Fitness in Childhood and Adolescence: A Powerful Marker of Health. Int. J. Obes..

[11] Stodden D., Sacko R., Nesbitt D. (2017). A Review of the Promotion of Fitness Measures and Health Outcomes in Youth. Am. J. Lifestyle Med..

[12] Ganley K.J., Paterno M.V., Miles C., Stout J., Brawner L., Girolami G., Warren M. (2011). Health-Related Fitness in Children and Adolescents. Pediatr. Phys. Ther..

[13] Drenowatz C., Greier K. (2018). Resistance Training in Youth—Benefits and Characteristics. J. Biomed..

[14] Smith J.J., Eather N., Morgan P.J., Plotnikoff R.C., Faigenbaum A.D., Lubans D.R. (2014). The Health Benefits of Muscular Fitness for Children and Adolescents: A Systematic Review and Meta-Analysis. Sports Med..

[15] Torres-Costoso A., López-Muñoz P., Martínez-Vizcaíno V., Álvarez-Bueno C., Cavero-Redondo I. (2020). Association Between Muscular Strength and Bone Health from Children to Young Adults: A Systematic Review. Sports Med..

[16] Bermejo-Cantarero A., Álvarez-Bueno C., Martínez-Vizcaino V., Redondo-Tébar A., Pozuelo-Carrascosa D.P., Sánchez-López M. (2021). Relationship between Both Cardiorespiratory and Muscular Fitness and Health-Related Quality of Life in Children and Adolescents: A Systematic Review and Meta-Analysis of Observational Studies. Health Qual. Life Outcomes.

[17] Park S.W., Yoon S.H., Lee S.M. (2024). Exploring the Relationship between Fundamental Movement Skills and Health-Related Fitness among First and Second Graders in Korea: Implications for Healthy Childhood Development. Healthcare.

[18] García-Hermoso A., Izquierdo M., Ramírez-Vélez R. (2022). Tracking of Physical Fitness Levels from Childhood and Adolescence to Adulthood: A Systematic Review and Meta-Analysis. Transl. Pediatr..

[19] Breau B., Brandes M., Veidebaum T., Tornaritis M., Moreno L.A., Molnár D., Lissner L., Eiben G., Lauria F., Kaprio J. (2022). Longitudinal Association of Childhood Physical Activity and Physical Fitness with Physical Activity in Adolescence: Insights from the IDEFICS/I.Family Study. Int. J. Behav. Nutr. Phys. Act..

[20] Sigmundsson H., Haga M. (2016). Motor Competence Is Associated with Physical Fitness in Four- to Six-Year-Old Preschool Children. Eur. Early Child. Educ. Res. J..

[21] Cattuzzo M.T., dos Santos Henrique R., Ré A.H.N., de Oliveira I.S., Melo B.M., de Sousa Moura M., de Araújo R.C., Stodden D. (2016). Motor Competence and Health Related Physical Fitness in Youth: A Systematic Review. J. Sci. Med. Sport.

[22] Okuda E., Horii D., Kano T. (2005). Genetic and Environmental Effects on Physical Fitness and Motor Performance. Int. J. Sport Health Sci..

[23] Venetsanou F., Kambas A. (2010). Environmental Factors Affecting Preschoolers’ Motor Development. Early Child. Educ. J..

[24] Huang W., Luo J., Chen Y. (2022). Effects of Kindergarten, Family Environment, and Physical Activity on Children’s Physical Fitness. Front. Public Health.

[25] Bergera S.E., Nuzzo K. (2008). Older Siblings Influence Younger Siblings’ Motor Development. Infant. Child. Dev..

[26] Peyre H., Albaret J., Bernard J.Y., Hoertel N., Melchior M., Forhan A., Taine M., Heude B., De Agostini M., Galéra C. (2019). Developmental Trajectories of Motor Skills during the Preschool Period. Eur. Child. Adolesc. Psychiatry.

[27] McHale S.M., Updegraff K.A., Whiteman S.D. (2012). Sibling Relationships and Influences in Childhood and Adolescence. J. Marriage Fam..

[28] Derikx D.F.A.A., Kamphorst E., van der Veer G., Schoemaker M.M., Hartman E., Houwen S. (2021). The Relationships between Sibling Characteristics and Motor Performance in 3-to 5-Year-Old Typically Developing Children. Int. J. Environ. Res. Public Health.

[29] Page M.J., McKenzie J.E., Bossuyt P.M., Boutron I., Hoffmann T.C., Mulrow C.D., Shamseer L., Tetzlaff J.M., Akl E.A., Brennan S.E. (2021). The PRISMA 2020 Statement: An Updated Guideline for Reporting Systematic Reviews. BMJ.

[30] Cheng S.Y., Wang T.T., Tai H.L. (2025). The Impact of Different Family Background on Children’s Fundamental Movement Skills Proficiency. BMC Public Health.

[31] Chiva-Bartoll O., Estevan I. (2019). Gender, Family Environment and Leisure Physical Activity as Associated Factors with the Motor Coordination in Childhood. A Pilot Study. RICYDE Rev. Int. Cienc. Del. Deport..

[32] Cruise S., O’Reilly D. (2014). The Influence of Parents, Older Siblings, and Non-Parental Care on Infant Development at Nine Months of Age. Infant. Behav. Dev..

[33] González-Devesa D., López-Eguía A., Amoedo L., Ayán-Pérez C. (2024). Associations between Agility, the Relative Age Effect, Siblings, and Digit Ratio (D2:D4) in Children and Adolescents. Children.

[34] González-Devesa D., Diz-Gómez J.C., Vicente-Vila P., Fernández M.D., Rodríguez M.R., Carballo-Afonso R., Sanchez-Lastra M.A., Ayán-Pérez C. (2025). Associations Between Relative Age, Siblings, and Motor Competence in Children and Adolescents. Children.

[35] Hayashida K., Nakatsuka M. (2014). Promoting Factors of Physical and Mental Development in Early Infancy: A Comparison of Preterm Delivery/Low Birth Weight Infants and Term Infants. Environ. Health Prev. Med..

[36] Jia C., Yang Z., Xin T., Li Y., Wang Y., Yang T. (2022). Differences in School Performance Between Only Children and Non-Only Children: Evidence From China. Front. Psychol..

[37] Krombholz H. (2006). Physical Performance in Relation to Age, Sex, Birth Order, Social Class, and Sports Activities of Preschool Children. Percept. Mot. Ski..

[38] Krombholz H. (2023). Motor Development of First Born Compared to Later Born Children in the First Two Years of Life—A Replication. Heliyon.

[39] Lopes V.P., Monteiro D. (2021). Socio-Cultural and Somatic Factors Associated with Children’s Motor Competence. J. Funct. Morphol. Kinesiol..

[40] Rebelo M., Serrano J., Duarte-Mendes P., Paulo R., Marinho D.A. (2020). Effect of Siblings and Type of Delivery on the Development of Motor Skills in the First 48 Months of Life. Int. J. Environ. Res. Public Health.

[41] Rodrigues L.P., Lima R.F., Silva A.F., Clemente F.M., Camões M., Nikolaidis P.T., Rosemann T., Knechtle B. (2020). Physical Fitness and Somatic Characteristics of the Only Child. Front. Pediatr..

[42] Rodrigues L.P., Luz C., Cordovil R., Mendes R., Alexandre R., Lopes V.P. (2021). Siblings’ Influence on the Motor Competence of Preschoolers. Children.

[43] Sáez-Sánchez M.B., Gil-Madrona P., Martínez-López M. (2021). Psychomotor Development and Its Link with Motivation to Learn and Academic Performance in Early Childhood Education. Rev. Educ..

[44] Šerbetar I., Peharda P., Plečko A. (2021). Differences in Physical Fitness and Body Measures Between Children with and Without Older Siblings. SKY-Int. J. Phys. Educ. Sport. Sci..

[45] Schild C.E., Meigen C., Kappelt J., Kiess W., Poulain T. (2022). Associations between Sociodemographic and Behavioural Parameters and Child Development Depending on Age and Sex: A Cross-Sectional Analysis. BMJ Open.

[46] Zareian E., Saeedi F., Rabbani V. (2014). The Role of Birth Order and Birth Weight in the Balance of Boys Aged 9-11 Years Old. Ann. Appl. Sport Sci..

[47] National Heart Lung and Blood Institute Quality Assessment Tool for Observational Cohort and Cross-Sectional Studies. https://www.nhlbi.nih.gov/health-topics/study-quality-assessment-tools.

[48] Cares-Marambio K., Montenegro-Jiménez Y., Torres-Castro R., Vera-Uribe R., Torralba Y., Alsina-Restoy X., Vasconcello-Castillo L., Vilaró J. (2021). Prevalence of Potential Respiratory Symptoms in Survivors of Hospital Admission after Coronavirus Disease 2019 (COVID-19): A Systematic Review and Meta-Analysis. Chron. Respir. Dis..

[49] Xiao E., Qin H., Zhu X., Jin J. (2023). The Influence of Birth Order and Sibling Age Gap on Children’s Sharing Decision. Early Child. Dev. Care.

[50] Havron N., Ramus F., Heude B., Forhan A., Cristia A., Peyre H. (2019). The Effect of Older Siblings on Language Development as a Function of Age Difference and Sex. Psychol. Sci..

[51] Alekseeva O.S., Kozlova I.E., Baskaeva O.V., Pyankova S.D. (2014). Intelligence and Sibling Relationship. Procedia-Soc. Behav. Sci..

[52] Bohn C., Vogel M., Poulain T., Hiemisch A., Kiess W., Körner A. (2022). Having Siblings Promotes a More Healthy Weight Status-Whereas Only Children Are at Greater Risk for Higher BMI in Later Childhood. PLoS ONE.

[53] Falbo T., Lin S., Garcia-Alexander G., Poston J.D.L. (2022). Sibling Effects on the Development of Obesity. International Handbook of the Demography of Obesity.

[54] Mackenzie K., Andronikos G., Travlos A., Souglis A., Fountain H., English C., Martindale R.J.J. (2022). Relationship between Sibling Characteristics and Talent Development. J. Phys. Educ. Sport.

[55] Delfa-de-la-Morena J.M., Pinheiro Paes P., Júnior F.C., Feitosa R.C., Lima de Oliveira D.P., Mijarra-Murillo J.-J., García-González M., Riquelme-Aguado V. (2025). Relationship of Physical Activity Levels and Body Composition with Psychomotor Performance and Strength in Men. Healthcare.

[56] Hopwood M.J., Farrow D., MacMahon C., Baker J. (2015). Sibling Dynamics and Sport Expertise. Scand. J. Med. Sci. Sport..

[57] Raudsepp L., Viira R. (2000). Influence of Parents’ and Siblings’ Physical Activity on Activity Levels of Adolescents. Eur. J. Phys. Educ..

[58] Mercê C., Branco M., Catela D., Lopes F., Rodrigues L.P. (2021). Cordovil, RLearning to Cycle: Are Physical Activity and Birth Order Related to the Age of Learning How to Ride a Bicycle?. Children.

[59] Keenan K., Barclay K., Goisis A. (2023). Health Outcomes of Only Children across the Life Course: An Investigation Using Swedish Register Data. Popul. Stud..

[60] Pantke P.M., Herrmann-Lingen C., Rothenberger A., Poustka L., Meyer T. (2023). Is Only-Child Status Associated with a Higher Blood Pressure in Adolescence? An Observational Study. Eur. J. Pediatr..

[61] Armstrong N., Barker A.R. (2011). Endurance Training and Elite Young Athletes. Med. Sport Sci..

[62] Barnett L., Hinkley T., Okely A.D., Salmon J. (2013). Child, Family and Environmental Correlates of Children’s Motor Skill Proficiency. J. Sci. Med. Sport.

[63] Honrubia-Montesinos C., Gil-Madrona P., Losada-Puente L. (2021). Motor Development among Spanish Preschool Children. Children.

[64] Fathirezaie Z., Matos S., Khodadadeh E., Clemente F.M., Badicu G., Silva A.F., Sani S.H.Z., Nahravani S. (2022). The Relationship between Executive Functions and Gross Motor Skills in Rural Children Aged 8–10 Years. Healthcare.

